# The complete mitochondrial genome of the Siberian Rubythroat (*Calliope calliope*) from Maorshan, China

**DOI:** 10.1080/23802359.2022.2163201

**Published:** 2023-01-09

**Authors:** Nan Zhang, Yao Zhao, Dehuai Meng, Xiaoyu Zhou, Zhensheng Liu, Liwei Teng

**Affiliations:** a College of Wildlife and Nature Reserve, Northeast Forestry University, Harbin, China; b The Siberian Tiger Park, Harbin, China; c Key Laboratory of Wildlife Conservation, State Forestry and Grassland Administration, Harbin, China

## Abstract

The Siberian Rubythroat (*Calliope calliope*), a shy and cautious passerine bird, is widely distributed throughout Siberia, Asia, and Europe. In this study, the complete mitogenome of the Siberian Rubythroat samples collected in Heilongjiang, China, was sequenced. The whole length of the complete mitochondrial genome is 16,840 bp, consisting of 13 protein-coding genes, 22 tRNA genes, two rRNA genes, and one control region. Only one overlapping gene was found among the 13 protein-coding genes (ND4L/ND4). The length of the control region is 1068 bp. The nucleotide composition is 29.32% A, 23.05% T, 14.80% G, and 32.82% C. Phylogenetic analysis indicated a close genetic relationship between *C. calliope* and *Luscinia cynaura*.

## 1. Introduction

### 1.1. Background

The Siberian Rubythroat (*Calliope calliope* Pallas 1776) is a small insectivorous bird that is widely distributed throughout Siberia and the Russian Far East (Ivanitskii and Monakhova 2020). Because of its melodious song, *C. calliope* is a valuable cage bird in China. The genome of *C. calliope* has not been published so far.

### 1.2. Justification

*C. calliope* is listed as Near Threatened according to the Red List of Threatened Species. The destruction of habitats preferred by these birds across their range is assumed to be the main cause of its uneven distribution and population decline. Moreover, the introduction of non-native species and climate change may threaten its survival. To better conserve this species, the complete mitogenome of *C. calliope* was sequenced.

## 2. Materials

The complete mitochondrial genome of *C. calliope* was sequenced using muscle tissue collected from samples in Heilongjiang, China. The specimens were deposited with the College of Wildlife and Protected Area, Northeast Forestry University, Harbin, China (No. BYQY201004; Zhensheng Liu: zhenshengliu@163.com). *C. calliope*, a member of the Muscicapidae family, selects forested habitats dominated by Erman’s birch and dwarf Siberian pine. They prefer shrub areas of low height that provides good cover (Xin et al. 2002).

## 3. Methods

Total genomic DNA was extracted and sequenced using a REPLI-g Mitochondrial DNA Kit on the Illumina Hiseq sequencing platform by Nanjing Personal Gene Technology Co. Ltd., China (Ru et al. 2021). High quality reads were then *de novo* assembled using MitoZ (Meng et al. 2019). The mitotic genome was annotated using the MITOS web server (Bernt et al. 2013). The phylogenetic relationship was inferred using the Neighbor-Joining method (Saitou and Nei 1987), performed in MEGA11 (Kumar et al. 2016). The bootstrap consensus tree, inferred from 1,000 replicates, was used to represent the evolutionary history of analyzed taxa (Felsenstein 1985).

## 4. Results and discussion

The mitochondrial genome is composed of 13 protein-coding genes, 2 rRNA genes (s-rRNA and l-rRNA), 22 tRNA genes, and 1 control region. The whole genome has a length of 16,840 bp, with a base composition of 29.32% A, 23.05% T, 14.80% G, and 32.82% C. The 13 protein-coding genes have a length of 10,997 bp, and all genes are encoded on the same strand, except for *ND6*, which is encoded on the light strand. All seven protein-coding genes start with ATG (*COX3*, *ND1*, *ND3*, *ND4*, *ND4L*, *ATP6*, and *ATP8*) except for *ND2*, *ND5*, and Cytb (ATC start codon), *ND6* (TAA start codon), and both *COX1* and *COX2* (GTG start codon). The total length of all tRNA genes is 1544 bp, ranging from 66 bp (tRNA-Ser and tRNA-Cys) to 76 bp (tRNA-Trp). The lengths of the two rRNA genes and the control region are 985 bp (s-rRNA), 1599 bp (l-rRNA), and 1068 bp (control region). The phylogenetic tree indicates that *C. calliope* is most closely related to *Luscinia cynaura*.

**Figure 1.**
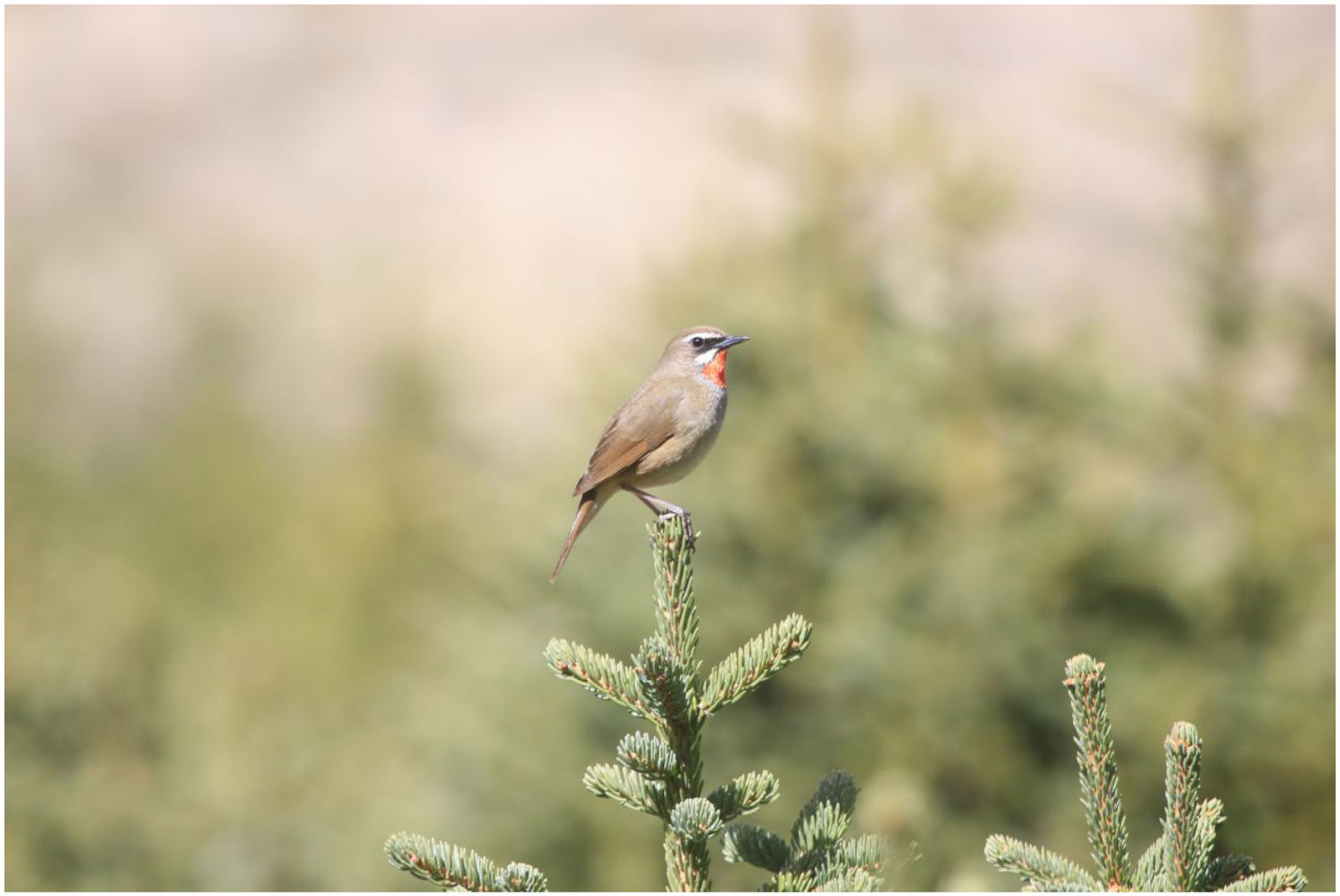
*Calliope calliope* reference image.

**Figure 2.**
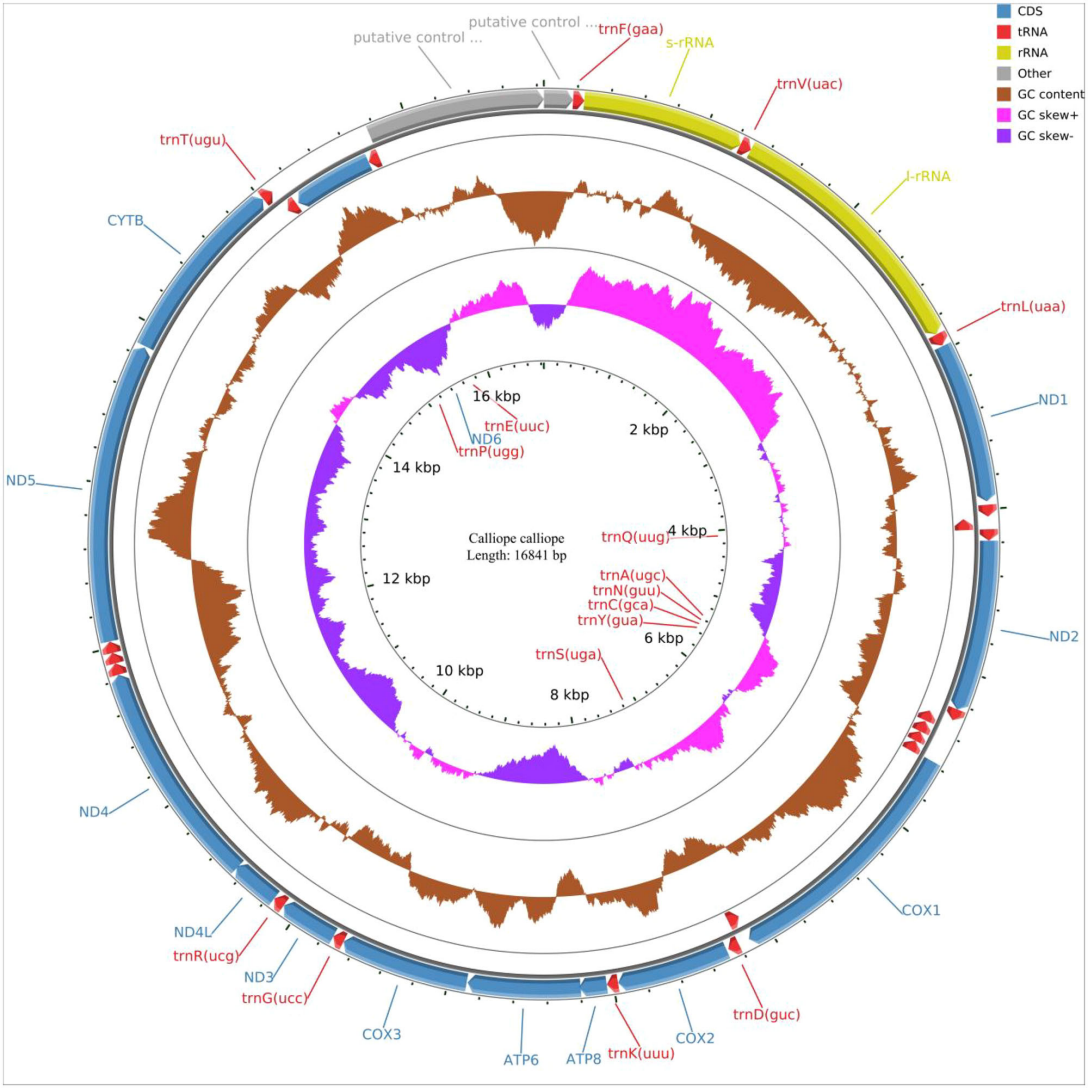
The complete mitochondrial genome map of *Calliope calliope*, including 13 protein-coding genes, 2 rRNA genes, 22 tRNA genes and 1 control region.

**Figure 3.**
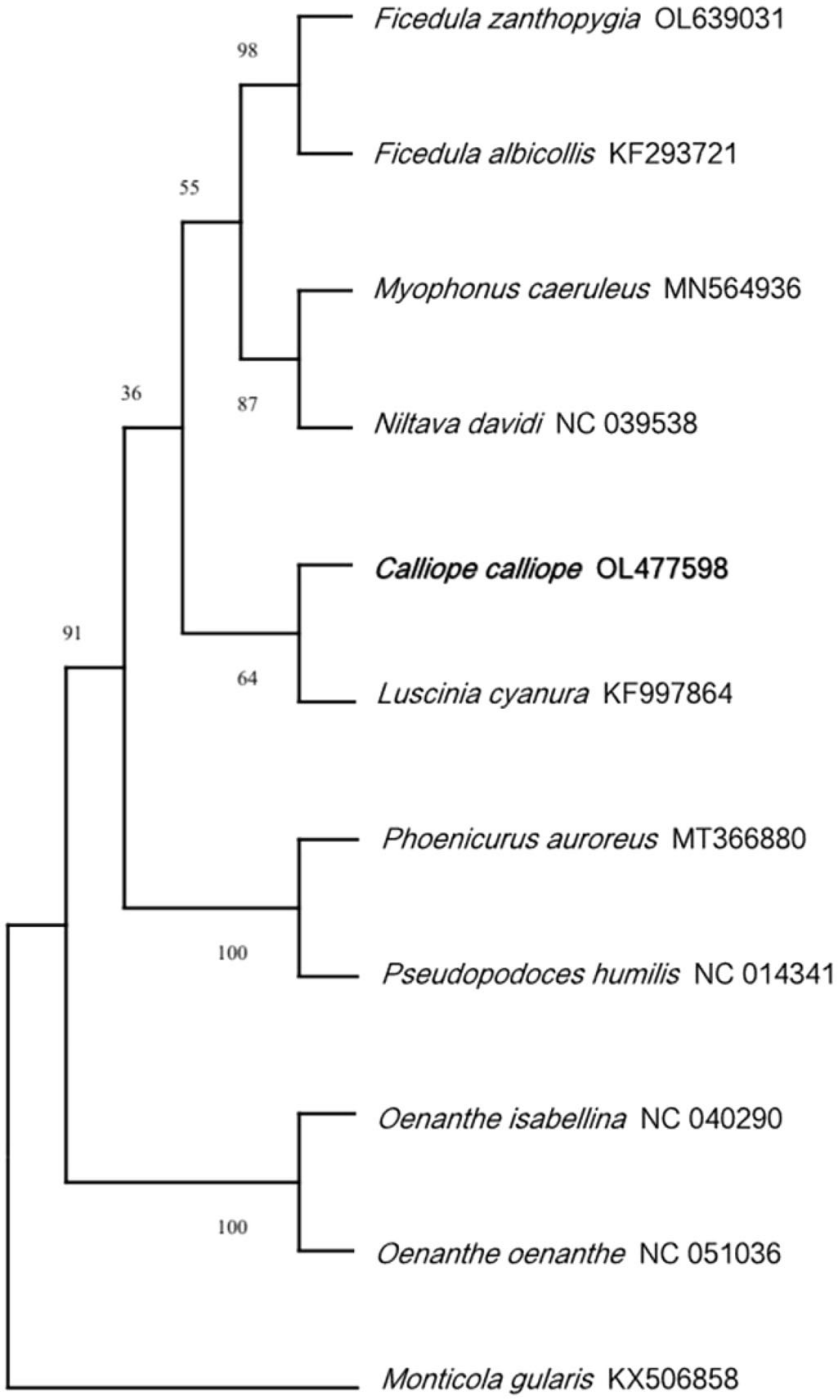
Phylogenetic tree generated using the Neighbor-Joining method based on the complete mitochondrial genomes of 11 species.

In conclusion, the complete mitochondrial genome of *C. calliope* provides valuable information for further genetic studies, thus contributing to improvements of conservation and management of *C. calliope* (Figures 1–3).

## Ethical approval

The muscle tissue of the Siberian Rubythroat was extracted from the individual which died naturally in the wild not more than three days old. Ethical approval is not required in this case.

## Data Availability

The data that support the findings of this study are openly available in NCBI at https://www.ncbi.nlm.nih.gov/, reference number OL477598. The associated BioProject, SRA, and Bio-Sample numbers are PRJNA781062, SRR16962849, and SAMN23227665, respectively.
